# Ordered hydroxyls on Ca_3_Ru_2_O_7_(001)

**DOI:** 10.1038/s41467-017-00066-w

**Published:** 2017-06-20

**Authors:** Daniel Halwidl, Wernfried Mayr-Schmölzer, David Fobes, Jin Peng, Zhiqiang Mao, Michael Schmid, Florian Mittendorfer, Josef Redinger, Ulrike Diebold

**Affiliations:** 1 Institute of Applied Physics, TU Wien, Wiedner Hauptstrasse 8-10/134,, 1040 Vienna, Austria; 2 Center for Computational Materials Science, TU Wien, Wiedner Hauptstrasse 8-10/134, A-1040 Vienna, Austria; 3 Department of Physics and Engineering Physics, Tulane University, New Orleans, LA 70118 USA

## Abstract

As complex ternary perovskite-type oxides are increasingly used in solid oxide fuel cells, electrolysis and catalysis, it is desirable to obtain a better understanding of their surface chemical properties. Here we report a pronounced ordering of hydroxyls on the cleaved (001) surface of the Ruddlesden-Popper perovskite Ca_3_Ru_2_O_7_ upon water adsorption at 105 K and subsequent annealing to room temperature. Density functional theory calculations predict the dissociative adsorption of a single water molecule (*E*_ads_ = 1.64 eV), forming an (OH)_ads_ group adsorbed in a Ca-Ca bridge site, with an H transferred to a neighboring surface oxygen atom, O_surf_. Scanning tunneling microscopy images show a pronounced ordering of the hydroxyls with (2 × 1), c(2 × 6), (1 × 3), and (1 × 1) periodicity. The present work demonstrates the importance of octahedral rotation and tilt in perovskites, for influencing surface reactivity, which here induces the ordering of the observed OH overlayers.

## Abstract (web summary)

As ternary perovskite-type oxides are increasingly used in fuel cells and catalysis, greater understanding of their surface chemical properties is required. Here the authors report a pronounced ordering of hydroxyls on the cleaved (001) surface of Ca_3_Ru_2_O_7_ perovskite induced by O-octahedral rotation and tilt.

## Introduction

Complex ternary oxides are intensively investigated for use in electrocatalytic reactions such as water splitting or the oxygen reduction reaction^1, 2^. Of particular interest are perovskite-type oxides, with the basic formula ABO_3_ (where A stands for an alkali, alkaline earth, or rare earth metal, while B refers to transition metal) and variations of this structure, such as double-perovskites, A_2_BB O_6_ or the Ruddlesden-Popper series A_n+1_B_n_O_3n+1_^3^. Microscopic insights into the interaction of perovskite-type oxides with relevant molecules, in particular with water, are sorely needed for progress in this area.

Surface science can deliver such insight as was shown for metal^4^ and metal-oxide surfaces^5^. The very first and fundamental question is whether a molecule in direct contact with the surface will adsorb as an intact entity or dissociate. This is governed by the subtle energy differences between interaction of water with the surface-cation on the one side and H bonding to surface O atoms on the other, and was addressed for several binary transition metal oxides. Molecular adsorption was found for water on anatase TiO_2_(101)^6, 7^ and on FeO(111)^8^. On TiO_2_(110)^9, 10^ and RuO_2_(110)/Ru(0001)^11, 12^ water adsorbs molecularly as well, but an equilibrium between molecular and short-lived dissociated states is under debate^12^. Mixed molecular and dissociative adsorption was observed on Fe_3_O_4_(001)^13^, Fe_3_O_4_(111)^13^ and ZnO(10-10)^14^. Water was found to exclusively dissociate on the (1 × 1) and (2 × 1) surfaces of α-Fe_2_O_3_(012)^15^ as well as α-Fe_2_O_3_(0001)^13^.

The AO-terminated surfaces of perovskites are closely related to the (001) surfaces of the binary alkaline earth oxides. On MgO(001) water partially dissociates and forms two stable structures with c(4 × 2) or p(3 × 2) symmetry, depending on temperature^16^. On CaO(001) mixed dissociative and molecular adsorption were experimentally observed already at very low coverages^17^. In a density functional theory (DFT) study Hu et al.^18^ derived two key factors that facilitate the dissociation of water on alkaline earth oxides: (i) an increase in the lattice constant, which enhances hydrogen bonding with the substrate, and (ii) the flexibility of the substrate. In a recent study^19^ on the SrO-terminated surfaces of the layered perovskites Sr_3_Ru_2_O_7_ and Sr_2_RuO_4_ it was shown that the water monomer adsorption also follows these key factors. Water dissociates into a (OH)_ads_, adsorbed on a cation-cation bridge site, and a proton that forms a surface hydroxyl with a surface oxygen atom. The (OH)_ads_ stays trapped by the surface hydroxyl, circling it by jumping between the four adjacent Sr-Sr bridge positions. The dynamic behavior of these ion pairs was predicted by theory^20^.

The rotation and tilting of the O octahedra in perovskites have been shown to influence ferroelectricity, magnetism, and electronic structure^21–24^, and these structural elements are key for understanding the physical properties of these complex materials. There are indications that this concept influences the structure/property relationship in solid-state chemistry as well—for example in the context of fuel cell materials, octahedral tilting and distortion was found to facilitate inter-octahedral proton transfer^25^ and O diffusion^26^, respectively, in the bulk. As motion of the octahedra renders inter-atomic distances inequivalent, this should result in different adsorption geometries and strengths of adsorbed molecules, and thus also be a decisive factor in surface chemistry.

Ca_3_Ru_2_O_7_, the prototypical perovskite material considered here, is a layered perovskite of the Ruddlesden-Popper series (alternating ABO_3_ and AO layers) and cleaves easily between adjacent CaO layers. The RuO_6_ octahedra are rotated in the *ab* plane and tilted by 12.9° with respect to the *c* axis. This affects the position of the surface O atom with respect to the four surrounding Ca-Ca bridge positions on the CaO terminated surface, rendering these sites inequivalent for water adsorption. This paper shows that the distance between a bridge site and its neighboring surface oxygen atoms is a pivotal factor for the water adsorption. Distinct ordering of the dissociated water is observed which can be fully rationalized with the surface geometry formed by the underlying octahedra. Based on accompanying DFT calculations, detailed structural models for the (2 × 1), (1 × 3), and (1 × 1) OH overlayers are presented.

## Results

### The pristine surface

**Fig. 1:**
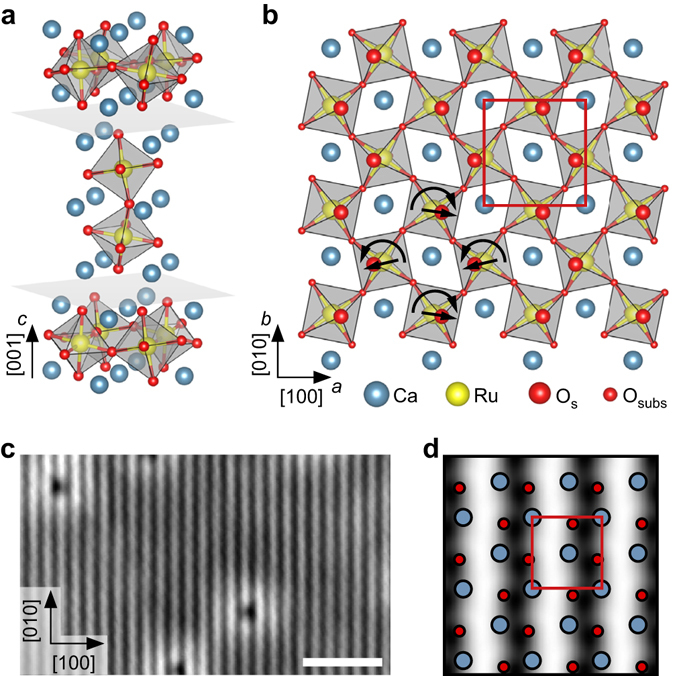
The cleaved calcium ruthenate surface. **a** Unit cell of the *n* = 2 member of the Ca_n+1_Ru_n_O_2n+1_ Ruddlesden Popper series. The crystal cleaves easily between neighboring CaO layers (marked by *gray* *planes*). **b** *Top view* of the CaO-terminated Ca_3_Ru_2_O_7_(001) surface. The RuO_6_ octahedra are alternately tilted with respect to the *c* axis (most pronounced in the *ac* plane), and rotated in the *ab* plane as indicated by the *straight* and *curved arrows*, respectively. The *red box* marks the orthorhombic unit cell (*a* = 5.365 Å, *b* = 5.562 Å). **c** STM image of the cleaved surface. The *dark* (*bright*) lines along the [010] direction correspond to areas where the apical oxygen atoms of the RuO_6_ octahedra are tilted toward (away from) each other (see panel **d**). The point defects are attributed to spurious impurities (see SI). The scale bar corresponds to 3 nm. STM parameters: *T*_sample_ = 78 K, *V*_sample_ = +0.8 V, *I*_tunnel_ = 0.1 nA; image rotated and cropped; fast scan direction is 68° clockwise from horizontal. **d** Tersoff-Hamann simulation of the cleaved surface

As a sample, Ca_3_Ru_2_O_7_ was chosen. This is the *n* = 2 member of the Ruddlesden-Popper series Ca_n+1_Ru_n_O_3n+1_, which consists of two perovskite-like CaRuO_3_ layers separated by adjacent CaO layers along the [001] direction (orthorhombic unit cell: *a* = 5.365 Å, *b* = 5.562 Å, *c* = 19.525 Å; see Supplementary Table 1). The RuO_6_ octahedra are alternately tilted with respect to the *c* axis, and alternately rotated in the *ab* plane^27^, where the *a*, *b*, and *c* axes correspond to the [100], [010], and [001] directions, respectively, see Fig. 1a, b. The preferred cleaving plane is between two CaO layers, with a cleaving energy of 3.62 eV (DFT value) per unit cell, or 0.91 eV per Ca-O bond. This low cleavage energy is consistent with experiments, where only the CaO termination is consistently observed. Terraces are typically at least a few hundred nm in size. Higher-resolution scanning tunneling microscopy (STM) images show alternating bright and dark lines along the [010] direction, see Fig. 1c. The dark (bright) lines correspond to areas where the apical oxygen atoms of the RuO_6_ octahedra are tilted toward (away from) each other, see Fig. 1d. The calculated creation energies for oxygen and calcium vacancies, 3.9 and 5.3 eV, respectively, are significantly higher than the cleaving energy per broken Ca-O bond. The point defects observed in the STM images thus are attributed to impurities (Ti, Sr, Mg, Ba) in the material (see Supplementary Note 1 and Supplementary Table 2) rather than intrinsic defects that would stem from the cleaving process.

### Water adsorption

**Fig. 2:**
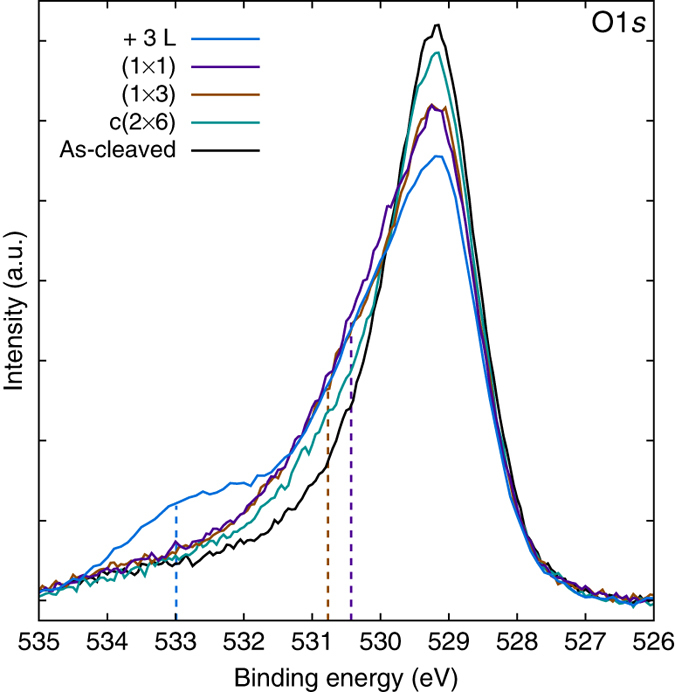
O1*s* XPS spectra of the as-cleaved surface and various OH overlayers observed by STM. Coverages up to and including the full monolayer lead to a shoulder between 530.0 and 531.0 eV BE for the c(2 × 6), (1 × 3) and (1 × 1) OH overlayer (full monolayer). Exposing the full monolayer to water at 105 K leads to a peak at 533.1 eV BE (labeled ‘+3 L’). In agreement with DFT calculations (*dashed vertical lines*, see Supplementary Table 3), the shoulder and the peak are assigned to hydroxyls and molecular water, respectively

First, increasing coverages of water are followed in the O1*s* X-ray photoelectron spectroscopy (XPS), see Fig. 2. The pristine surface exhibits an asymmetric peak at 529.2 eV binding energy (BE), broadened towards the high BE side. According to DFT calculations the O1*s* core level for the oxygen atoms in the CaO plane is shifted to 0.7 eV higher BE compared to the RuO_2_ planes which serve as DFT reference. Keeping in mind that the higher BE O1*s* signal of the CaO planes contributes less to the total O1*s* intensity, the observation of only one asymmetric peak in the experiment is attributed to the limited resolution of the experimental setup. For all annealed water structures observed by STM (i.e., c(2 × 6), (1 × 3) and (1 × 1), see below) the spectrum shows a shoulder between 530.0 and 531.0 eV BE; i.e., roughly 1.5 eV above the as-cleaved O peak. This fits well to the calculated BE shifts of 1.57 and 1.23 eV for surface hydroxyls in the (1 × 3) and (1 × 1) OH overlayer, respectively (see Supplementary Table 3). Only after saturating the surface with hydroxyls and dosing additional water at 105 K without annealing, a peak at 533.1 eV BE is observed; i.e., 3.9 eV above the bulk oxygen peak. This fits well to the calculated BE shift of 3.63 eV for molecular water. Therefore, in agreement with the DFT results, the shoulder and the peak are attributed to dissociated and molecular water, respectively.

### The water monomer

**Fig. 3:**
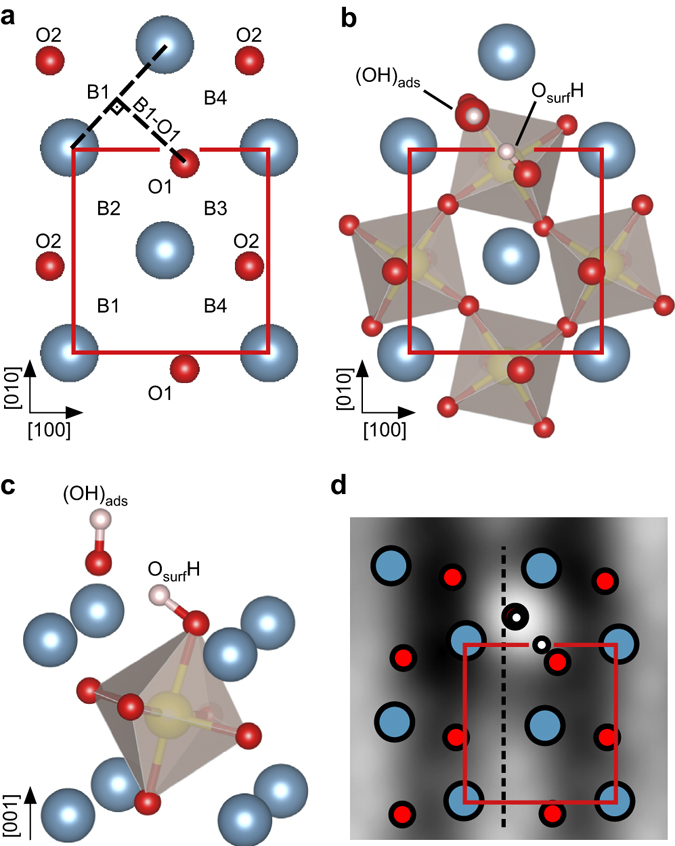
DFT model for water adsorption on cleaved Ca_3_Ru_2_O_7_. **a** *Top view* DFT model of the pristine surface (Ca—*blue*, O—*red*, H—*white*, Ru—*yellow*). The unit cell is marked by the *rectangle*. The distances from the bridge sites B1 to B4 to the surface oxygen atoms O1 and O2 differ because of the tilted RuO_6_ octahedra and are listed in Table 1. The distance B1-O1 is shown as an example, other distances are constructed analogously. **b** *Top view* and **c** *side view* of the DFT model of the most preferred configuration B1O1 for one dissociated water monomer with *E*_ads_B1O1_ = 1.64 eV. The first subsurface layer of RuO_6_ octahedra is shown. Water dissociates, forming an (OH)_ads_ group adsorbed on Ca-Ca bridge site B1 with a H transferred to the neighboring surface oxygen atom, O1. The tilt angle of the hydroxylated RuO_6_ octahedron increases from 12.9° to 16.3°, when compared to the pristine surface. The energetically equivalent configuration B2O2 is not shown. **d** According to Tersoff-Hamann simulations, STM shows the (OH)_ads_ as a bright feature. When compared to the Ca-Ca bridge centre (*dashed line*), the (OH)_ads_ sits slightly closer to the O_surf_H (in [100] direction)

Figure 3a shows a DFT model of the pristine surface with inequivalent adsorption sites labeled as surface oxygen atoms (O_surf_) O1 and O2, and Ca-Ca bridge sites B1 to B4. The inequivalence is caused by the rotation and tilting of the RuO_6_ octahedra, leading to different distances between the O_surf_ and the bridge sites, see Table 1. The calculations predict that a single water molecule dissociates without a barrier, forming an (OH)_ads_ group adsorbed on a bridge site, with the split-off H transferred to a neighboring O_surf_. The adsorption energy depends on which bridge site the (OH)_ads_ fragment is placed with respect to the O_surf_. If the split-off H is adsorbed on O1, the (OH)_ads_ is clearly preferred to sit on B1 (named B1O1 configuration) with an overall adsorption energy of *E*_ads_B1O1_ = 1.64 eV, see Fig. 3b, c. Compared to the pristine surface the tilt angle of the hydroxylated RuO_6_ octahedron increases from 12.9° to 16.3°. The adsorption energy, O-Ru-O bond angle and O-O distances between the (OH)_ads_ and the O_surf_ for all configurations are listed in Table 2. The adsorption geometries for the configurations are shown in Supplementary Fig. 1. The configuration B4O1 is not stable, resulting in diffusion of the (OH)_ads_ to the most preferred B1 site. If the split-off H is adsorbed on O2, the preferred bridge site is B2 as the same calculation applies, but for symmetry reasons the roles of B1 and B3 switch with B2 and B4, respectively.

**Table 1:** Bridge site to O_surf_ distances for the pristine surface

Bridge-O1	Distance (Å)	Bridge-O2	Distance (Å)
B1	2.56	B1	1.98
B2	1.98	B2	2.56
B3	1.29	B3	1.90
B4	1.90	B4	1.29

The distances differ because of the rotated and tilted RuO_6_ octahedra (see Figs. 1 and 3)

**Table 2:** Adsorption configuration details

Configuration	*E*_ads_	O–Ru–O	OH_ads_-O1	OH_ads_-O2	Slab size	H_2_O molecules	Coverage
	(eV)	(°)	(Å)	(Å)	(surface unit cells)		(ML)
B1O1	1.64	177.6	2.56	3.0	3 × 3	1	0.06
B2O1	1.44	176.2	2.52	3.18	3 × 3	1	0.06
B3O1	0.78	161.9	2.50	2.81	3 × 3	1	0.06
(2 × 1)	1.62	–	–	–	2 × 1	2	0.5
c(2 × 6)	–	–	–	–	–	–	0.58
(1 × 3)	1.59	–	–	–	1 × 3	4	0.67
(1 × 1)	1.48	–	–	–	1 × 1	2	1.0
Molecular	0.78	–	–	–	1 × 1	1	0.5

The adsorption energies for the dissociated monomer configurations decrease with increasing distortion of the hydroxylated octahedron, reflected in the decreasing O–Ru–O bond angle along the octahedron's *c* axis (rows 1–3). The O–O distance between the OH_ads_ and the O_surf_ is similar for all configurations. The adsorption energy of the OH overlayers monotonically decreases with increasing coverage (rows 4 to 7)

In the optimum adsorption position B1O1, the adsorption energy of the dissociated molecule (*E*_ads_ = 1.64 eV) is significantly higher than for the intact molecule (*E*_ads_ = 0.78 eV). The Ca-O distance of the (OH)_ads_ fragment is 2.39 Å. The OH bond length of 0.97 Å for the (OH)_ads_ fragment is slightly smaller than the value of 1.03 Å found for the O_surf_H bond. The latter bond is tilted which allows the formation of an additional hydrogen bond (O-O distance 2.57 Å, classified as strong hydrogen bond^4^) to the (OH)_ads_ fragment, with an H-(OH)_ads_ distance of 1.55 Å. The (OH)_ads_ does not sit centred on the bridge site but slightly closer to its respective O_surf_H. A Tersoff-Hamann (TH) simulation shows the (OH)_ads_ as a bright feature, see Fig. 3d.

**Fig. 4:**
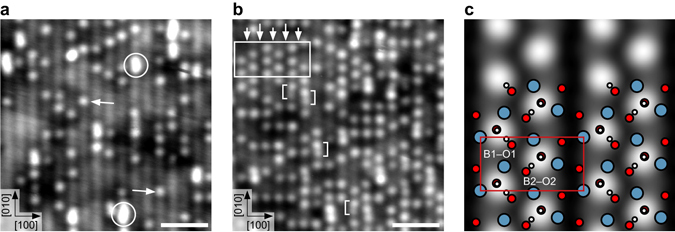
Water adsorbed on cleaved Ca_3_Ru_2_O_7_ and the (2 × 1) OH overlayer. **a** STM image of 0.3 Langmuir water dosed at 105 K. Single, bright features (*arrows*, 0.13 ML) that sit on the bright lines of the substrate are attributed to the (OH)_ads_ of dissociated water. This agrees with the model that predicts the (OH)_ads_ to adsorb on bridge sites B1 or B2, i.e., on the bright lines of the substrate (see Fig. 3). The larger, brighter features (*circles*) contain several H_2_O molecules. **b** STM image of the sample in panel **a** after annealing for 1 h at room temperature. The coverage increase from 0.13 to 0.34 ML corroborates that the larger, bright features (*circled* in panel **a**) contained several H_2_O molecules that became mobile, dispersed across the surface and dissociated. The *box marks* a more densely covered area with local (2 × 1) symmetry. Double lines are formed, with a smaller gap within the double line, and a wider gap separating two double lines, marked by *short* and *long arrows*, respectively. See panel **c** for model. The *brackets* mark spots separated by half a unit cell along [010], named bright-dark feature. This feature is tentatively assigned to two (OH)_ads_ on neighboring bridge sites, i.e., B1-O1 and B2-O2, with one of the (OH)_ads_ appearing darker than the other. **c** DFT model and TH simulation of the (2 × 1) OH overlayer, corresponding to a coverage of 0.5 ML. The single-unit-cell spacing along each line leads to occupation of either the B1O1 or the symmetrically equivalent B2O2 configuration (see Fig. 3). In adjacent lines the (OH)_ads_ are shifted by half a unit cell in [010] direction and thus occupy the other configuration. Therefore, the (OH)_ads_ on adjacent lines are shifted along [100] in opposite directions, leading to narrow and wide gaps between the lines. STM parameters: *T*_sample_ = 78 K, *V*_sample_ = −0.8 V, *I*_tunnel_ = 0.1 nA; fast scan direction is 18° clockwise from horizontal. All scale bars correspond to 2 nm

STM images show single bright features after exposing the as-cleaved sample to 0.3 Langmuir (L, where 1 L equals an exposure to 1 × 10^−8^ mbar for 133 s) of water at 105 K, see Fig. 4a. These features are separated by at least one unit cell and sit exclusively on top of the bright substrate lines. According to the DFT model and TH simulations (see Fig. 3d) these bright features are (OH)_ads_. Annealing the sample for 1 h at room temperature leads to an apparent coverage increase from 0.13 monolayer (ML, with respect to two adsorption sites per unit cell) to 0.34 ML, see Fig. 4b. This substantial increase suggests that the larger features, labeled with an oval in Fig. 4a, contained several H_2_O molecules that became mobile, dispersed across the surface, and dissociated.

### OH overlayers

The white box in Fig. 4b marks a densely covered area, where six neighboring substrate lines are occupied. This results in a local (2 × 1) symmetry. The (OH)_ads_ along the same substrate line are spaced one unit cell apart from each other. On the neighboring line the (OH)_ads_ are shifted by half a unit cell along the [010] direction. Double lines are formed, with a smaller gap within one double line, and a wider gap separating two double lines. The gaps are explained considering the preferred adsorption sites discussed in the context of Fig. 3. Only the most favorable configurations are occupied, i.e., B1O1 in one line and the symmetrically equivalent B2O2 in the adjacent lines. This causes the (OH)_ads_ in adjacent lines to shift in opposite directions along [100], and leads to the narrow and wide gaps between the lines. Figure 4c shows the DFT model and the TH simulation of the (2 × 1) OH overlayer, corresponding to a 0.5 ML coverage. The calculation gives an adsorption energy decrease of 20 meV per H_2_O molecule compared to the isolated water monomer, suggesting slightly repulsive interaction between the (OH)_ads_ (see Table 2).

The brackets in Fig. 4b mark spots separated by half a unit cell along [010], named bright-dark feature. The feature is tentatively assigned to two (OH)_ads_ on neighboring bridge sites, i.e., B1O1 and B2O2, with one of the (OH)_ads_ appearing darker than the other. Interestingly, the darker (OH)_ads_ are all oriented toward the [0–10] direction.

**Fig. 5:**
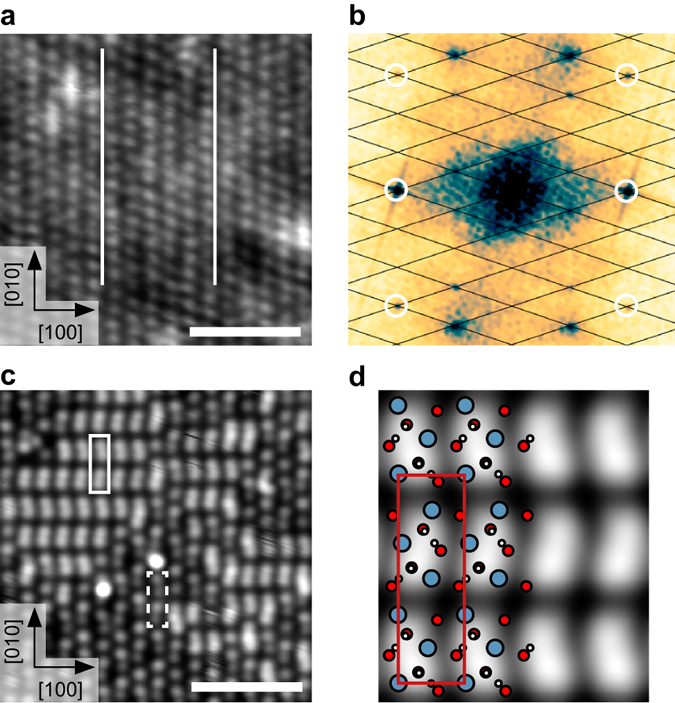
The c(2 × 6) and (1 × 3) OH overlayer. **a** STM image of 0.5 L water dosed at 110 K and subsequently annealed for 1 h at room temperature. The (OH)_ads_ along [010] are not perfectly straight but slightly undulate (marked by lines to guide the eye). STM parameters: *T*_sample_ = 78 K, *V*_sample_ = −0.4 V, *I*_tunnel_ = 0.2 nA; fast scan direction is 18° clockwise from horizontal. **b** Fourier transform of the STM image in panel **a**. The grid shows the primitive unit cell of the OH overlayer; the *circles mark* the substrate spots. The overlayer exhibits a c(2 × 6) periodicity corresponding to a coverage of 0.58 ML (7/6 H_2_O per unit cell). **c** STM image after exposing the c(2 × 6) OH overlayer to 0.15 L at 100 K and annealing at room temperature for 1 h. The surface is covered by domains of a (1 × 3) OH overlayer (*solid box*) as well as a mixture of bright-dark features (*dashed box*) and water monomers STM parameters: *T*_sample_ = 78 K, *V*_sample_ = −0.8 V, *I*_tunnel_ = 0.1 nA; fast scan direction is 18.5° clockwise from horizontal. **d** DFT model and TH simulation of the (1 × 3) OH overlayer, corresponding to 0.67 ML. Along [010] pairs of dissociated water in the most favorable configurations are formed, i.e., B1O1 and B2O2 (shown here), or B2O2 and B1O1, with one unoccupied bridge site between them. The *box marks* the superstructure cell, which spans three substrate unit cells in [010] direction. All scale bars correspond to 3 nm

Increasing the initial dose to 0.5 L at 100 K, and annealing for 1 h at room temperature leads to a c(2 × 6) OH overlayer that covers the whole surface (see Fig. 5a, b). The OH overlayer is now compressed along [010]; 7 (OH)_ads_ are found on six unit cells, with a shift by half an OH-OH distance between adjacent lines. The (OH)_ads_ along [010] do not form perfectly straight lines, but slightly undulate. The coverage increase from 0.34 to 7/12 = 0.58 ML (again, with respect to O_surf_ or Ca sites) fits well to the dose increase from 0.3 to 0.5 L when compared to the results in Fig. 4b.

**Fig. 6:**
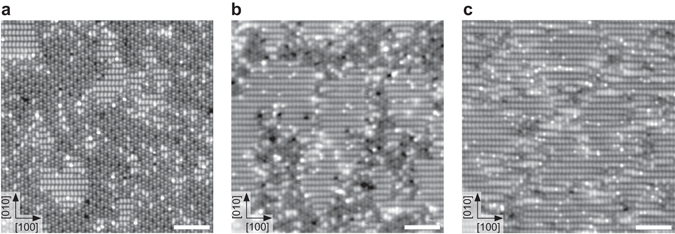
Effect of increasing water coverage. STM images after exposing the c(2 × 6) OH overlayer to increasing water doses at 105 K and decreasing annealing times at room temperature. **a** 0.15 L and 1 h annealing time. **b** 0.5 L and 30 min annealing time. **c** 1.0 L and 15 min annealing time. The higher the overall water coverage, the more surface is covered by the (1 × 3) OH overlayer. In panel **c** it covers almost the whole surface. STM parameters: *T*_sample_ = 78 K, *V*_sample_ = −0.8 V, *I*_tunnel_ = 0.1 nA; fast scan direction is 18° clockwise from horizontal. All scale bars correspond to 5 nm

Exposing the c(2 × 6) OH overlayer to an additional H_2_O dose of 0.15 L at 100 K, and annealing for 1 h at room temperature results in a (1 × 3) OH overlayer corresponding to 0.67 ML, see Fig. 5c. The surface is covered by (1 × 3) domains surrounded by a mixture of the bright-dark features and single water monomers. According to the DFT model and TH simulation, the (1 × 3) OH overlayer consists of dimers of dissociated water in the most favorable configurations along [010], i.e., B1O1 and B2O2 (see Fig. 5d), or B2O2 and B1O1, with one unoccupied bridge site between them. The bright-dark features are separated by one unoccupied bridge site from either another bright-dark feature or a water monomer (along [010]). Counting the number of bright-dark features and water monomers in a large scale STM image (Fig. 6a) gives an average 0.60 ML coverage for the mixture.

Further increasing the water coverage by increasing the initial dose to the c(2 × 6)OH overlayer and decreasing the annealing time results in the (1 × 3) domains covering first an increasing part and then almost the whole surface, see Fig. 6b, c, respectively. The experimental annealing procedure (i.e., placing the sample into a RT environment for 15 min) leaves considerable uncertainty regarding the exact temperature that was reached; probably it was somewhere between 160 and 300 K.

**Fig. 7:**
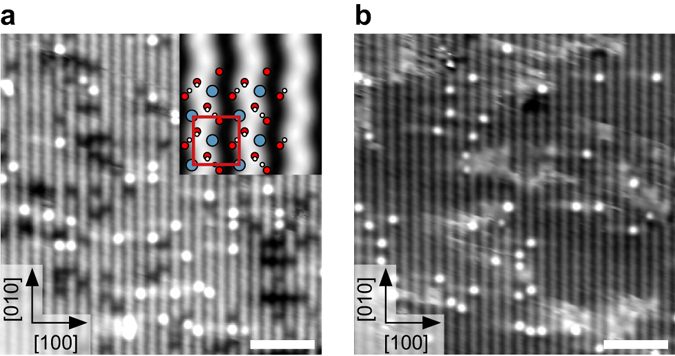
The (1 × 1) OH overlayer and molecular water. **a** STM image after dosing 0.4 L on the almost fully-developed (1 × 3) overlayer in Fig. 7c. The sample is almost completely covered by undulating, bright lines in [010] direction, showing only a few gaps, where the (1 × 3) structure is still visible. The single, bright features are unidentified adsorbates (approx. 0.03 ML) from the residual gas. The *inset* shows the DFT model and TH simulation of the (1 × 1) OH overlayer, i.e., the full monolayer. Both equivalent adsorption sites (B1O1 and B2O2) in the unit cell are occupied, i.e., all surface oxygens are hydroxylated. **b** STM image after exposing the sample in panel **a** to 0.75 L water. The gaps in the undulating bright hydroxyl lines have closed. The diffuse, *white patches* are assigned to molecular water (see Fig. 2). STM parameters: *T*_sample_ = 78 K, *V*_sample_ = −0.8 V, *I*_tunnel_ = 0.1 nA; fast scan direction is 18° clockwise from *horizontal*. All scale bars correspond to 3 nm

Dosing 0.4 L to the (1 × 3) covered surface leads to bright, slightly undulating lines along [010] with almost no gaps, see Fig. 7a. The continuous, undulating lines agree well with TH simulations for a (1 × 1) OH overlayer, i.e., the full monolayer with all O_surf_ atoms hydroxylated, see inset Fig. 7a. Dosing more water to the fully hydroxylated surface without annealing leads to diffuse, white patches in STM that are assigned to molecular water moving under the tip, see Fig. 7b.

Annealing an OH covered sample for longer times at room temperature leads to partial desorption of water. Supplementary Fig.  2a shows the sample in Fig. 6c (i.e., almost fully covered by the (1 × 3) OH overlayer) after annealing for 3 h at room temperature. Only a few (1 × 3) OH overlayer patches remain while the sample is mainly covered by a mixture of the (2 × 1) OH overlayer and bright-dark features. A different OH covered sample annealed for 20 min at 330 K was still covered by patches of the (2 × 1) OH overlayer, see Supplementary Fig. 2b. A rough estimate for the adsorption energy based on the desorption rates and temperatures is 1 to 1.26 eV. Annealing at higher temperatures was not possible because of the epoxy glue that was used to mount the sample.

### Adsorption at oxygen vacancies

**Fig. 8:**
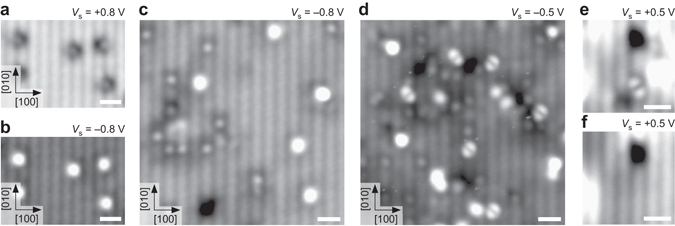
Oxygen vacancies on cleaved Ca_3_Ru_2_O_7_. **a** Empty and **b** filled-states STM image of Ca_3_Ru_2_O_7_ after irradiation with 1 keV electrons at 105 K. In empty states, the oxygen vacancy (*V*_O_) appears as a small, *bright dot* on the *dark substrate* line with a one-sided, *dark patch* extending into the bright substrate line. In filled states, the *V*_O_ appears as a large, *bright dot* on the *dark substrate* line. **c** Filled-states STM image after exposure to 0.05 L water. **d** Different area than in panel **c** and after annealing to room temperature for 50 min. The *bright*, *double-lobed* features are attributed to neighboring surface hydroxyls, formed by the dissociation of water that filled the *V*_O_. **e** Small-area STM image of a *double-lobed* feature next to unidentified adsorbates. **f** Same area as in panel **e**, but after scanning the area with a bias of +4.5 V. The *double-lobed* feature has desorbed and at its former location the pristine surface is left behind. STM parameters: *T*_sample_ = 78 K, *I*_tunnel_ = 0.1 nA, for *V*_sample_ see figures; fast scan direction is 16° **a**–**d** and 18° **e**–**f** anticlockwise from horizontal. All scale bars correspond to 1 nm

Oxygen vacancies (*V*_O_) were investigated as they are a common theme in metal-oxides and especially important for mixed ionic and electronic conductors, serving as electrodes in solid oxide fuel cells^3, 28^. *V*_O_s do not form spontaneously when cleaving the samples; here they were instead created by irradiating the clean surface by 1 keV electrons^29, 30^, see Fig. 8a, b. At low temperatures no interaction was observed between the water and the *V*_O_ (Fig. 8c), but after annealing to room temperature bright, double-lobed features are observed that sit centred on the bright substrate lines (Fig. 8d). These features are assigned to two neighboring surface hydroxyls, formed by the dissociation of water that filled the *V*_O_. The adsorption energy for dissociative adsorption at an oxygen vacancy is 2.41 eV according to the DFT calculations. The hydrogen can be desorbed by scanning with a high positive bias voltage^31^ leaving behind the pristine surface (Fig. 8e, f).

## Discussion

On the basis of our XPS experiments and DFT calculations it is clear that water adsorbs dissociatively on Ca_3_Ru_2_O_7_. STM images show ordering of the (OH)_ads_ in agreement with the calculated adsorption model. Interestingly, the evolution of ordered structures, which at first sight is rather complex, can be rationalized by considering the rotation and tilt of the O octahedra. In the most preferred configuration B1O1 (Fig. 3b) the tilt of the hydroxylated octahedron increases from 12.9° to 16.3° towards its natural tilting direction when the water dissociates at this site. The O-Ru-O angle changes minimally from 178.2° to 177.6°. The O-O distances between the (OH)_ads_ and O1 and O2 are 2.57 and 2.98 Å, respectively. The weaker adsorption energies for the configurations B2O1 and B3O1 correlate foremost with the forced tilting of the octahedron that is necessary to accommodate the dissociation fragments at similar O-O distances; in both cases this tilt goes against the natural direction and distorts the O-Ru-O angles (see Table 2 and Supplementary Fig. 1). The order of adsorption preference (B1O1, B2O1, B3O1) is in agreement with the decreasing distances between the bridge site and O1 on the pristine surface, which, in turn, are an indicator for the natural tilting direction of the octahedron (see Table 1).

With the largest bridge site to O_surf_ distance (i.e., B1-O1 and B2-O2) providing the highest adsorption energy only these sites are filled and, at low coverages, every other bridge site along [010] is left out, leading to a local (2 × 1) ordering of individual hydroxide pairs (Fig. 4b). As the coverage increases, the (2 × 1) OH overlayer is compressed along [010] and forms the c(2 × 6) OH overlayer with 7 (OH)_ads_ on six unit cells (0.58 ML). The undulation probably arises from the inequivalent positions of the (OH)_ads_ with respect to the bridge site due to the compression. Additionally, the protons of the (OH)_ads_ may be oriented differently depending on the exact positions of the oxygens. Further increasing coverage leads to fragments of the dissociated water locating at neighboring bridge sites in the (1 × 3) OH overlayer and the bright-dark features (see Fig. 5c). The ratio of surface covered by the (1 × 3) OH overlayer to surface covered by the mixture of bright-dark features and water monomers depends on the overall water coverage. The more water, the more surface is covered by the (1 × 3) OH overlayer (see Fig. 6). This trend agrees with the (1 × 3) OH overlayer being equivalent to 0.67 ML and the mixture of bright-dark features and monomers being equivalent to roughly 0.60 ML. The skipping of available sites and the monotonic decrease in adsorption energy as the coverage increases (see Table 2) suggests that the ordering is governed by electrostatic repulsion. On BaO(001)^32^ the electrostatic repulsion between (OH)_ads_ and O_surf_H was shown to be screened by the Ba^2+^cations. However, the polarizability^33^ of Ca^2+^ is four times lower than of Ba^2+^, and Ca_3_Ru_2_O_7_(001) is less symmetric than BaO(001) thus the screening may be less effective.

The bright-dark feature is suggested to be a dissociated water dimer in two adjacent bridge sites along [010], eventually leading to a full (1 × 3) OH overlayer. However, in the precursor bright-dark structure the (OH)_ads_ that is oriented toward the [0-10] direction appears darker. A possible explanation is the inequivalence of the bridge sites available for the second water once the first water has adsorbed and dissociated. In one case the (OH)_ads_ is next to the unoccupied site, in the other case the O_surf_H. However, DFT calculations resulted in no energy differences for the two cases.

It is instructive to compare the adsorption behavior on Ca_3_Ru_2_O_7_ to the related strontium ruthenate perovskite and the simpler CaO, where the surface is much more symmetric and where detailed surface measurements are available. The dissociation into an (OH)_ads_ fragment, adsorbed on a cation bridge site, and into a proton, adsorbed on a neighboring surface oxygen, was recently observed on the SrO-terminated surface of Sr_n+1_Ru_n_O_3n+1_ (*n* = 1,2)^19^. There the oxygen octahedra are not tilted with respect to the [001] direction, hence the surface oxygen sublattice is square and all bridge sites are equally spaced from the O_surf_. The (OH)_ads_ fragment hops between the four equivalent cation bridge sites around the O_surf_H fragment at liquid-nitrogen temperature. In the present work no hopping of the (OH)_ads_ was observed. This again agrees with one of the bridge sites surrounding the O_surf_ being clearly preferred for adsorption (see Table 2). At low coverages dimers are not formed, but every other bridge site along the [010] direction is unoccupied as discussed above. In contrast, on Sr_n+1_Ru_n_O_3n+1_ stable dimers in adjacent Sr-Sr bridge sites are preferentially formed, evolving into one-dimensional chains^19^. On the simpler binary oxide CaO(001) the dissociation of the water monomer was predicted with adsorption energies around 0.9 eV^18, 20^. The higher adsorption energy of 1.64 eV on Ca_3_Ru_2_O_7_ fits well to the reported key relevance of the lattice constant, as here the Ca-Ca distance is 10.2% larger than on CaO(001)^18^. Experimentally, mixed dissociative and molecular adsorption were observed already at very low coverages on CaO(001)^17^, in contrast to the exclusively dissociative adsorption on Ca_3_Ru_2_O_7_. It is interesting that O_surf_H hydroxyl pairs that form when water dissociates at an oxygen vacancy and fills it also show preferential arrangement. (Here such *V*_O_s were not observed on the as-cleaved surface but artificially created by electron bombardment.) After dosing water at 105 K and annealing to room temperature bright, double-lobed features formed (Fig. 8d). The two lobes always span a bright substrate line, indicating that the split-off proton prefers to adsorb on the O_surf_ that has the larger distance (4.5 vs. 3.2 Å) to the surface hydroxyl that formed at the location of the healed *V*_O_.

In conclusion, water adsorbs exclusively dissociatively on Ca_3_Ru_2_O_7_(001) upon water exposure at 105 K and subsequent annealing to room temperature. The resulting ordered OH overlayers are (except for the large-cell c(2 × 6) OH overlayer) fully rationalized with DFT calculations. Molecular water is not observed in any overlayer up to full monolayer coverage. The OH overlayers show a pronounced ordering of the dissociation fragments, caused by inequivalent adsorption sites in the surface unit cell. This inequivalence originates from the distorted oxygen sublattice consisting of the apical oxygen atoms of the underlying rotated and tilted RuO_6_ octahedra. The present work thus demonstrates the crucial influence of octahedral rotation and tilt on the surface reactivity of perovskites, suggesting that the engineering of these structural elements (e.g., by utilizing strain) could be useful for tuning the surface chemistry of these increasingly important materials.

## Methods

### Experimental set-up and sample preparation

The experiments were carried out in an ultra-high vacuum (UHV) system consisting of a preparation chamber and an STM chamber with base pressures of 2 × 10^−11^ and 6 × 10^−12^ mbar, respectively. A low-temperature STM (commercial Omicron LT-STM) was operated at 78 K in constant-current mode using an electro-chemically etched W-tip. The bias voltage was applied to the sample; positive or negative bias voltages result in STM images of the unoccupied or occupied states, respectively. All STM images shown were corrected for distortions as described elsewhere^34^. High-quality calcium ruthenate single crystals were grown by the floating zone technique using a mirror-focused furnace^35^. The composition of the samples was determined via inductively coupled plasma mass spectroscopy using laser ablation for direct analysis of the solid samples. Before insertion into the UHV, the samples were fixed on stainless-steel sample plates with conducting silver epoxy glue (EPO-TEK H21D, Epoxy Technology Inc.), and a metal stud was glued on top with another epoxy adhesive (EPO-TEK H77, Epoxy Technology Inc.). The crystals were cleaved in the analysis chamber at 100 K by removing the metal stub with a wobble stick (see Supplementary Note 2 for comment on success rate). Deionized water (Millipore water, purified in-house) was further cleaned by several freeze-pump-thaw cycles and was dosed in the preparation chamber while keeping the sample at 105 K. Annealing was done by bringing the sample in contact with an annealing stage, held at room temperature. For creating O vacancies, the sample was bombarded by electrons from a well-outgassed electron source in the preparation chamber with the sample held at 105 K. XPS was performed in the preparation chamber using non-monochromatized Mg Kα X-rays and a SPECS PHOIBOS 100 electron analyzer at normal emission with a pass energy of 15 eV.

### Density functional theory

The calculations were performed using the Vienna Ab-initio Simulation Package. This code employs the projector augmented plane wave formalism and PAW potentials^36^ with an energy cutoff of 400 eV. As a proper treatment of dispersion effects is important for the adsorption of water^37^ the so-called optB86b^38, 39^ functional was used. The k-point meshes used were generated by the Monkhorst-Pack^40^ scheme using a Gamma-centred 6 × 6 × 1 mesh for the surface calculations on the 1 × 1 unit cell and correspondingly reduced meshes for the larger cells. The structural relaxations were performed until the residual forces were below 0.01 eV/Å (see Supplementary Note 3). Slabs consisting of one double layer Ca_3_Ru_2_O_7_ separated by 15 Å of vacuum were used, similar to the model previously used for strontium ruthenate^19^. STM images were simulated using the TH^41^ approach and all core level shifts were calculated in the final state approximation^42^.

### Data availability

The data that support the findings of this study are available from the corresponding author on request.

## Electronic supplementary material

Peer Review File Supplementry Figs and TablesReviewer reports and authors' response from the peer review of this Article at Nature Communications

Supplementary Information Supplementry Figs and Tables
