# Supplementary material for: Ordered hydroxyls on Ca3Ru2O7(001)
Source: Nat Commun. 2017 Jun 20;8:23. doi: 10.1038/s41467-017-00066-w (PMC5478604; doi:10.1038/s41467-017-00066-w)
Supplement: Supplementary file 2 — Supplementry Figs and Tables [file 41467_2017_66_MOESM2_ESM.pdf]

File Name: Supplementary Information
Description: Supplementary Figures, Supplementary Tables, Supplementary Notes and Supplementary References.

File Name: Peer review file
Description:

Supplementary Note 1

The composition of two samples of the same batch was determined via inductively coupled plasma mass spectroscopy (ICP-MS) using laser ablation for direct analysis of the solid samples. The mass fractions of the found impurities (Ti, Sr, Ba and Mg) are listed in Supplementary Table 2. The samples had comparable impurity contents, the main impurities being Ti and Sr. This is consistent with the observation of point defects in STM images of as-cleaved samples. The amount of impurities observed by STM usually varies when measuring samples from different batches.

Supplementary Note 2

The sample cleaving was not always successful with regard to producing a flat, defect-free, atomically resolvable surface. The success rates were approximately 70 and 50 percent for old and new samples, respectively, where old and new mean storage times of approximately 4 and 1 years, respectively. Common outcomes were roughness on the nm-scale, clusters of unknown adsorbates ("dirt"), line defects and 2D defects. Some samples appeared non-conductive in STM. The cause for that may be a combination of sample degradation over storage time (humidity), varying impurity contents and cleaving along areas of aggregated impurities. Furthermore, the samples may have already developed unnoticed cracks along such areas while handling the ready-to-cleave assembly (sample plate, sample, metal stud) in the ambient.

Supplementary Note 3

The bulk structure was optimized using the van-der-Waals corrected optB86 functional. The lattice constants showed very good agreement with the experimental values (see Supplementary Table 1).

Supplementary Table 1: Comparison of the lattice constants between the optimized slab and experimental data.

Lattice constant	vdW-DF [pm]	Experiment (RT)[1] [pm]
a	536.5	537.8
b	556.2	552.3
c	1952.5	1958.7

Supplementary Table 2: Mass fractions of impurities measured by ICP-MS. Quantification by NIST612 and ^{44}Ca as internal standard.

Element		Sample 17			Sample 22		
Ti	[ppm]	135.6	±	0.20	135.3	±	0.39
Sr	[ppm]	79.9	±	1.43	80.1	±	0.77
Ba	[ppm]	4.4	±	0.19	4.2	±	0.13
Mg	[ppm]	13.4	±	0.27	14.6	±	1.12

Supplementary Table 3: O1*s* core level shifts of molecular and dissociated H_2O; referenced to the O1*s* level in the RuO_2 plane.

Slab size [unit cells]	H_2O molecules	Coverage [ML]	Shift [eV]	Configuration
1×1×0.5	1	0.5	3.79	molecular
3×3×0.5	1	0.06	1.77	B1O1
1×3×0.5	4	0.67	1.57	(1 × 3) OH overlayer
1×1×0.5	2	1.0	1.23	(1 × 1) OH overlayer

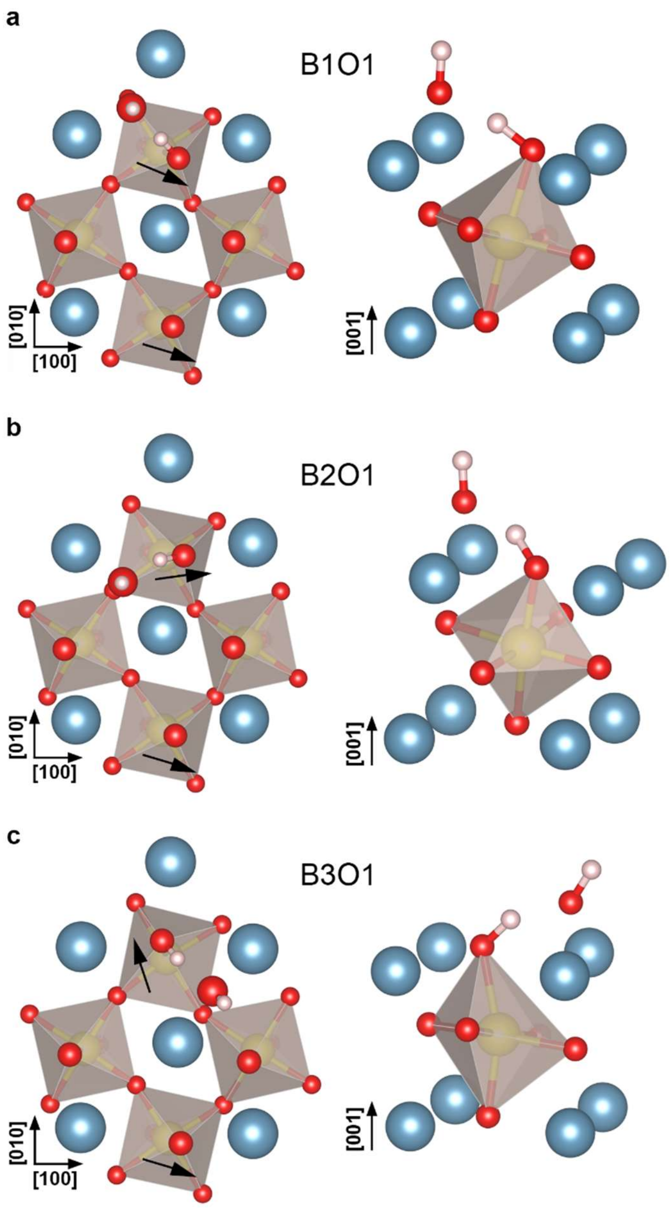

Supplementary Figure 1: Top and side view DFT models of the different monomer adsorption configurations. The arrows mark the tilt direction of the apical oxygen of the hydroxylated and regular octahedra. The decreasing adsorption energy (B1O1 > B2O1 > B3O1) follows the increasingly deviating tilt direction and the decreasing O-Ru-O bond angle (see Table 2).

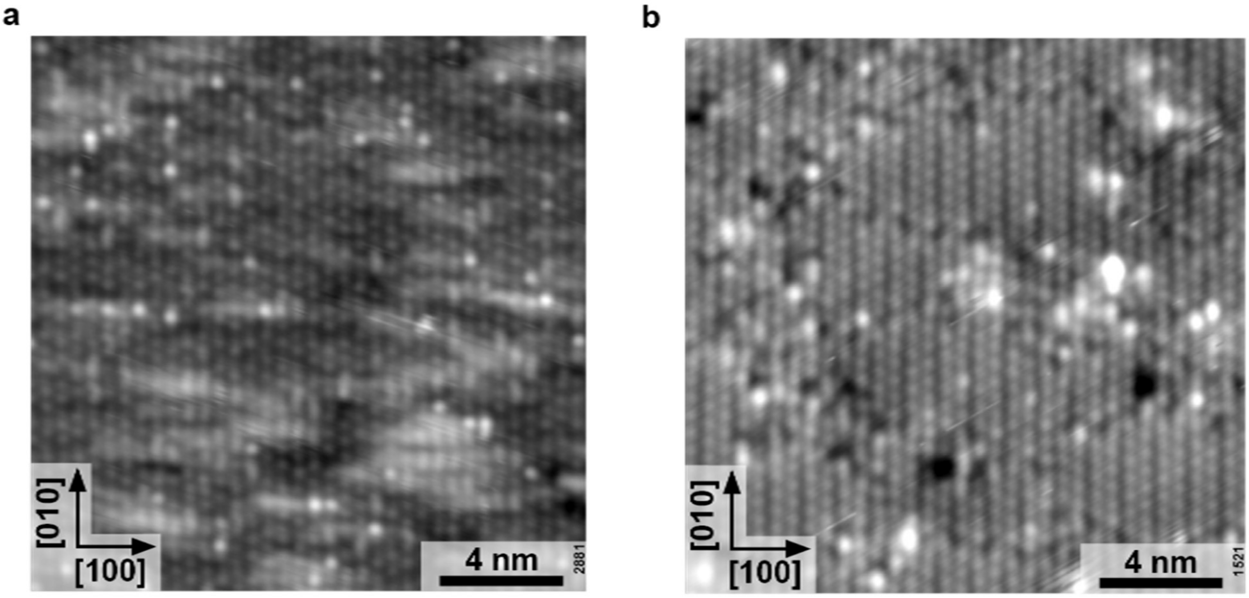

Supplementary Figure 2: Samples annealed to different temperatures.

(a) Sample as prepared in Figure 6c after annealing for 3 hours at room temperature. The water has partially desorbed and a few (1 × 3) OH overlayer patches remained on the surface that is mainly covered by a mixture of the (2 × 1) OH overlayer and bright-dark features. (STM parameters: T_{sample} = 78 K, V_{sample} = -0.8 V, I_{tunnel} = 0.1 nA; fast scan direction is 18° clockwise from horizontal.)

(b) A different OH covered sample after annealing for 20 minutes at 330 K. The sample is still covered by patches of the (2 × 1) OH overlayer. (STM parameters: T_{sample} = 78 K, V_{sample} = -0.8 V, I_{tunnel} = 0.1 nA; fast scan direction is 29° clockwise from horizontal.)

Supplementary References

1. Yoshida, Y. *et al.* Crystal and magnetic structure of $Ca_3Ru_2O_7$. *Phys. Rev. B* **72,** 54412 (2005).
