# Supplementary material for: Ordered hydroxyls on Ca3Ru2O7(001)
Source: Nat Commun. 2017 Jun 20;8:23. doi: 10.1038/s41467-017-00066-w (PMC5478604; doi:10.1038/s41467-017-00066-w)
Supplement: Supplementary file 1 — Supplementry Figs and TablesReviewer reports and authors' response from the peer review of this Article at Nature Communications [file 41467_2017_66_MOESM1_ESM.pdf]

Reviewers' comments:

Reviewer #1 (Remarks to the Author):

The manuscript by Diebold et. al reports for the first time on the adsorption of water on cleaved $Ca_3Ru_2O_7$ surfaces. The STM measurements show several well-order overlayers depending on the amount of water present. The XPS spectra reveal that water adsorbs exclusively dissociative on $Ca_3Ru_2O_7$ and therefore the ordered overlayers are OH structures. Experimental findings are supported by density functional theory calculations.

The experimental work seems to be well carried out and the DFT calculations carefully crafted. I suggest publication of the manuscript if the following points can be addressed by the authors:

• Page 4: line 78: "…with a cleaving energy of 3.62 eV…" and line 85 "…cleaving energy per broken Ca-O bond of 0.91 eV…", how do the two cleaving energies relate to each other?
• DFT shows that the dissociative adsorption is energetically favored. It would be informative to know also something about the dissociation barrier.
• Page 6 / Fig.4: The temperatures are not consistent: line 130 "105K" and line 484 "110K".
• The authors are suggested to comment whether there is a bias dependence in the imaging of the OH, H overlayers on the surface.

Reviewer #2 (Remarks to the Author):

The methods presented in the paper is of general interest, and the ordering of the hydroxyls on the $Ca_3Ru_2O_7$ surface is interesting. I would recommend publication after a minor revision of the paper. I have following comments:

1. The authors are working on a specific $Ca_3Ru_2O_7$ Ruddlesden-Popper material and not a general perovskite material, as they made clear on line 72 in the Results section. However, this point is not clearly stated in the Abstract and Introduction, where the authors talked entirely about perovskites and not mentioning the Ruddlesden-Popper series. This might be misleading to possible audiences, as the RP materials have a few unique properties that is not shared by ABO_3 perovskites. For example, the RP material can have two possible cleaved AO termination with different subsurface layers. I recommend the authors to explain the structural features of the RP materials (i.e. alternating ABO_3/AO layers) in the introduction and refer to the RP anisotropy in the abstract.

2. The water adsorption experiments are mainly carried out at cryogenic temperature (105 K) and annealed to room temperature later. However, for water-splitting or solid oxide fuel cell applications, the working temperature is usually room temperature or above. I understand that it might be difficult, but some comments on the relevance of the octahedral tilting to the water splitting reactivity at higher temperature will be very useful. For example, will the octahedral tilting change with respect to temperature and oxygen vacancy? And what will be the implications to the water adsorption?

3. The authors only presented DFT results of the water adsorption on the ideal $Ca_3Ru_2O_7$ (001) surface. I understand that no water adsorption at the surface oxygen vacancy sites is observed at 105 K, but it is very common for the water adsorption to take place at oxygen vacancies at room temperature or higher. The authors should add a comparison of the calculated water adsorption energy between the presented Ca-Ca site and the oxygen vacancy site, which is very relevant and of interest.

RESPONSE TO REFEREE REPORTS

Reviewer #1 (Remarks to the Author):

The manuscript by Diebold et. al reports for the first time on the adsorption of water on cleaved Ca3Ru2O7 surfaces. The STM measurements show several well-order overlayers depending on the amount of water present. The XPS spectra reveal that water adsorbs exclusively dissociative on Ca3Ru2O7 and therefore the ordered overlayers are OH structures. Experimental findings are supported by density functional theory calculations.

The experimental work seems to be well carried out and the DFT calculations carefully crafted. I suggest publication of the manuscript if the following points can be addressed by the authors:

• Page 4: line 78: "…with a cleaving energy of 3.62 eV…" and line 85 "…cleaving energy per broken Ca-O bond of 0.91 eV…", how do the two cleaving energies relate to each other?

> 3.62 eV is the cleaving energy per unit cell. Each unit cell contains 4 Ca-O bonds in the cleaving plane, hence the cleaving energy per Ca-O bond is 0.91 eV. To make that clear, the sentence was edited accordingly.

• DFT shows that the dissociative adsorption is energetically favored. It would be informative to know also something about the dissociation barrier.

> The calculations show that the dissociation proceeds without barrier. The sentence (line 114) was edited accordingly

• Page 6 / Fig.4: The temperatures are not consistent: line 130 "105K" and line 484 "110K".

> This typo was corrected.

• The authors are suggested to comment whether there is a bias dependence in the imaging of the OH, H overlayers on the surface.

> The $(OH)_{ads}$ fragment is shown as bright feature that sits on the bright lines of the substrate, independent from the value or polarity of the bias voltage. The $O_{surf}H$ fragment could never be observed, likely because the $(OH)_{ads}$ fragment is much higher than the $O_{surf}H$ (1.65 Å), so the STM contrast is dominated by geometry. However, negative bias on the sample (i.e., imaging filled states) resulted in better contrast than positive bias for all observed overlayers.

We thank the reviewer for his/her comments and the positive evaluation.

Reviewer #2 (Remarks to the Author):

The methods presented in the paper is of general interest, and the ordering of the hydroxyls on the $Ca_3Ru_2O_7$ surface is interesting. I would recommend publication after a minor revision of the paper. I have following comments:

1. The authors are working on a specific Ca3Ru2O7 Ruddlesden-Popper material and not a general perovskite material, as they made clear on line 72 in the Results section. However, this point is not clearly stated in the Abstract and Introduction, where the authors talked entirely about perovskites and not mentioning the Ruddlesden-Popper series. This might be misleading to possible audiences, as the RP materials have a few unique properties that is not shared by ABO3 perovskites. For example, the RP material can have two possible cleaved AO termination with different subsurface layers. I recommend the authors to explain the structural features of the RP materials (i.e. alternating ABO3/AO layers) in the introduction and refer to the RP anisotropy in the abstract.

We thank the reviewer for pointing this out. To make clear that $Ca_3Ru_2O_7$ is a layered perovskite, the abstract and introduction were edited accordingly.

2. The water adsorption experiments are mainly carried out at cryogenic temperature (105 K) and annealed to room temperature later. However, for water-splitting or solid oxide fuel cell applications, the working temperature is usually room temperature or above. I understand that it might be difficult, but some comments on the relevance of the octahedral tilting to the water splitting reactivity at higher temperature will be very useful. For example, will the octahedral tilting change with respect to temperature and oxygen vacancy? And what will be the implications to the water adsorption?

The temperature dependence of the structure of $Ca_3Ru_2O_7$ was studied by Yoshida et al.[1] using neutron scattering up to 292 K. Although they did not directly indicate how the tilting angle varies with temperature, their data of the temperature dependence of the Ru-O1-Ca2 bonding angle (see Figure on the right) appears

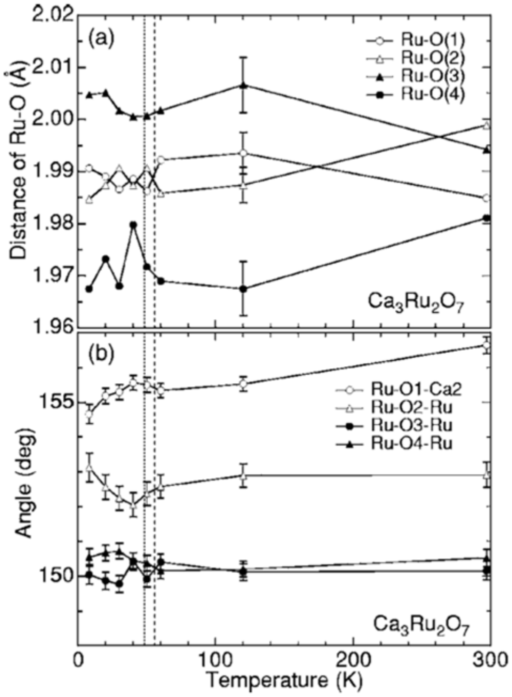

FIG. 6. Temperature dependences of (a) bond lengths of the RuO_6 octahedra and (b) angles between the octahedra in $Ca_3Ru_2O_7$. Solid lines are connected between the data points. The broken and doted lines denote the Néel temperature (56 K) and the structural transition temperature (48 K), respectively.

Reprinted figure with permission from Yoshida, Yoshiyuki and Ikeda, Shin-Ichi and Matsuhata, Hirofumi and Shirakawa, Naoki and Lee, C. H. and Katano, Susumu. Phys. Rev. B, 72, 54412 (2005). Copyright 2005 by the American Physical Society

to suggest that the tilting angle slightly decreases with increasing temperature.

In both Ca_2RuO_4 and $Ca_3Ru_2O_7$, the orthorhombic structure is caused by RuO_6 octahedral rotation and tilt. For Ca_2RuO_4, when both rotation and tilt disappear at elevated temperatures (> 357 K), its structure becomes tetragonal[2]. If $Ca_3Ru_2O_7$ follows a similar trend (this has not been studied), we would still expect dissociative water adsorption similar to the water adsorption on the tetragonal $Sr_{n+1}Ru_nO_{3n+1}$ (n = 1, 2)[3]. However, as water already partially desorbs at room temperature it is questionable if water would still adsorb at the elevated temperatures necessary for the structure change.

Regarding the tilt change with respect to an oxygen vacancy: in our DFT calculations we find only a very minor (<1.5°) tilting angle increase of the one nearest octahedron pointing towards the oxygen vacancy (octahedron labeled '1' in Figure and Table below) when compared to the tilting angle on the pristine surface of 13.11°. The tilting angle of the other 3 nearest octahedra virtually does not change (see Table). Therefore we do not expect significant implications of the tilting angle changes induced by oxygen vacancies for the adsorption of water.

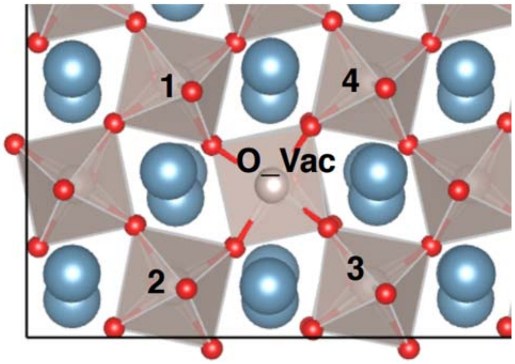

Octahedron	Tilting angle [°]
1	14.60
2	13.10
3	13.33
4	13.16

References

1. Yoshida, Y. *et al.* Crystal and magnetic structure of $Ca_3Ru_2O_7$. *Phys. Rev. B* **72,** 54412 (2005).

2. Alexander, C. *et al.* Destruction of the Mott insulating ground state of Ca_2RuO_4 by a structural transition. *Phys. Rev. B* **60,** R8422–R8425 (1999).

3. Halwidl, D. *et al.* Adsorption of water at the SrO surface of ruthenates. *Nat. Mater.* **15,** 450–456 (2015).

3. The authors only presented DFT results of the water adsorption on the ideal Ca3Ru2O7 (001) surface. I understand that no water adsorption at the surface oxygen vacancy sites is observed at 105 K, but it is very common for the water adsorption to take place at oxygen vacancies at room temperature or higher. The authors should add a comparison of the calculated water adsorption energy between the presented Ca-Ca site and the oxygen vacancy site, which is very relevant and of interest.

The adsorption energy (2.41 eV) for dissociative adsorption at the oxygen vacancy site was added to the oxygen vacancy paragraph at the end of the results section (line 205).

We thank the reviewer for his/her comments and his/her positive evaluation.

REVIEWERS' COMMENTS:

Reviewer #1 (Remarks to the Author):

The authors have thoroughly addressed my previous comments. Therefore, I recommend publication of this manuscript.

Reviewer #2 (Remarks to the Author):

The authors have addressed correctly all my comments. The paper can be published in this form.
